# Primary hyperoxaluria diagnosed after kidney transplantation: a case report and literature review

**DOI:** 10.1186/s12882-021-02546-0

**Published:** 2021-11-27

**Authors:** Zhitao Cai, Mao Ding, Rengui Chen, Jiefu Zhu, Lian Li, Xiongfei Wu

**Affiliations:** Center of Nephrology, Dialysis and Transplantation, Renmin Hospital of Wuhan University, No.238 Jiefang Road, Wuchang District, Wuhan, 430060 Hubei China

**Keywords:** Primary hyperoxaluria, Kidney transplantation, Acute rejection, Needle biopsy

## Abstract

**Background:**

Primary hyperoxaluria (PH) is a rare inherited autosomal recessive disease caused by disturbed glyoxylate metabolism. The disease is characterized by calcium oxalate crystal deposition in various organs, especially in the kidney. Due to the lack of current understanding of PH, nearly all patients are only initially diagnosed with PH when recurrent lithiasis and progressive end-stage renal disease occur. Many cases are not diagnosed in patients until renal allograft insufficiency occurs after renal transplantation. This case report and literature review aim to emphasize the need for careful pre-transplant PH screening of patients with bilateral nephrocalcinosis or nephrolithiasis.

**Case presentation:**

Renal allograft insufficiency was diagnosed as PH after kidney transplantation. Here, we detail the complete clinical course, including computed tomography images of the original kidney and renal graft, histopathological images of a biopsy of the transplanted kidney, the results of laboratory and molecular genetic tests, and the treatment. In addition, we reviewed the literature from 2000 to 2021 and analyzed 19 reported cases of PH diagnosed after kidney transplantation, and provide a summary of the characteristics, complications, treatment, and prognosis of these cases.

**Conclusions:**

By reviewing and analyzing these cases, we concluded that patients with a history of nephrocalcinosis or nephrolithiasis in both kidneys need preoperative screening for PH and appropriate treatment before kidney transplantation. Delayed graft function caused by PH is easily misdiagnosed as acute rejection, and needle biopsy should be performed at an early stage.

**Supplementary Information:**

The online version contains supplementary material available at 10.1186/s12882-021-02546-0.

## Background

Primary hyperoxaluria (PH) is a rare inherited autosomal recessive disease. Oxalate overproduction due to deficiencies of enzymes required for glyoxylate metabolism is the most common mechanism leading to this disease [1, 2]. According to different gene mutations, there are three types of PH. In primary hyperoxaluria, approximately 70% of cases are PH type 1 (PH1), in which an alanine-glyoxylate aminotransferase (AGXT) gene mutation occurs in liver peroxisomes. PH type 2 and PH type 3 each account for approximately 10% of all cases and are caused by mutations in the genes for glyoxylate reductase/hydroxy pyruvate reductase (GRHPR) and 4-hydroxy-2-oxoglutarate aldolase (HOGA), respectively. The pathogenesis of the remaining about 10% of cases is still unclear [3]. The excess oxalate can be deposited in various organs, especially in the kidney, in the form of calcium oxalate (CaOx) crystal. Lithiasis, nephrocalcinosis, and ultimately progression to end-stage renal disease (ESRD) are common manifestations of PH [4]. However, due to the lack of understanding of PH, patient history of primary renal calcification and stones is often ignored and the diagnosis of PH is not made until the loss of renal allograft function after kidney transplantation. This study introduces a case of PH with bilateral nephrocalcinosis leading to delayed graft function (DGF) of the renal graft, reviews and analyzes all reported cases of PH diagnosed after kidney transplantation from 2000 to 2021. This study aims to summarize the manifestations, complications, treatment, and prognosis of these cases and emphasize the need for careful pre-transplant PH screening of patients with bilateral nephrocalcinosis and nephrolithiasis.

## Case presentation

**Fig. 1:**
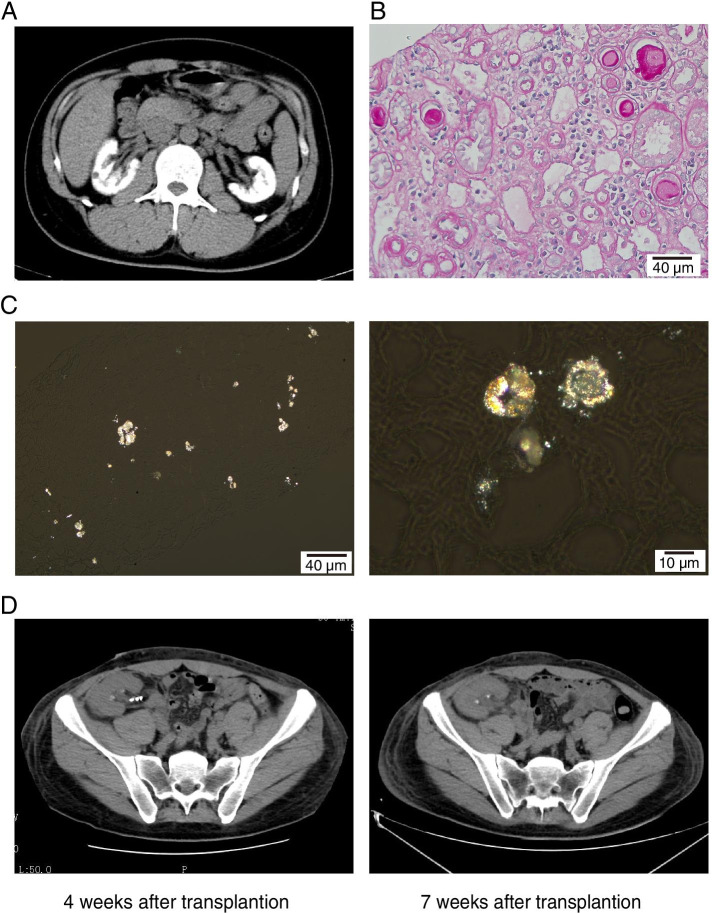
Clinical data of patient. **A** CT image of the kidneys showed that the patients had bilateral nephrocalcinosis. **B** Renal transplant biopsy on postoperative day 21 detected borderline acute rejection. (200× HE) **C** Renal transplant biopsy on postoperative day 21 detected extensive CaOx crystals deposition in the interstitial tubule. (Polarized light 200× and 400×) **D** CT examination demonstrated that CaOx crystals deposition of graft renal increased in several weeks after transplantation

A 32-year-old male hypertensive patient was hospitalized in our department on January 29, 2021, with a 6-month history of elevated serum creatinine (SCr), accompanied by hypertension (peak 180/100 mmHg), and a 5-month history of maintenance hemodialysis. Computed tomography (CT) examination revealed bilateral nephrocalcinosis (Fig. 1A). With preoperative SCr 1296.00 μmol/L and blood urea nitrogen (BUN) 20.90 mmol/L, the patient was scheduled to receive a kidney allograft from a deceased kidney donor followed by hypertension treatment and standard triple immunosuppression with mycophenolate mofetil, tacrolimus and methylprednisolone (Methylpred). Routine blood biochemical analysis and therapeutic drug monitoring were regularly undertaken as follow-up assessments. Unfortunately, the renal graft gradually developed DGF with SCr 674.00 μmol/L and BUN 46.34 mmol/L. Due to the suspicion of acute rejection, high doses of methylprednisolone were administered for 3 days. Ultrasonography-guided needle biopsy of the renal graft was performed on postoperative day 21.

**Fig. 2:**
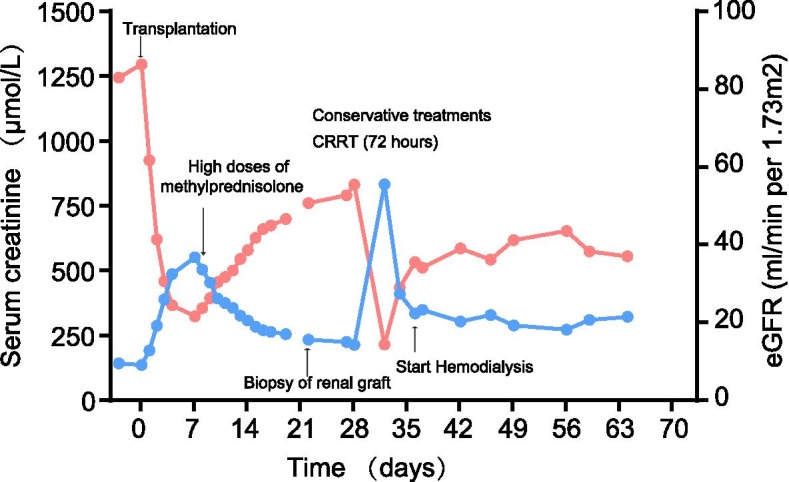
The variation trend of serum creatinine and eGFR and managements in the renal transplant recipient. Conservation treatments includes high fluid intake, avoidance of high oxalate foods, and vitamin B6

Borderline acute rejection and deposition of CaOx crystals in the allograft were detected on renal graft biopsy (Fig. 1B and C). The patient and his parents received molecular genetic testing and mutations in the AGXT gene were identified (1. exon 1/CDS1:c.120-121insC;p.Gly41Argfs*127 and 2. exon 4/CDS4:c.473C > T;p.Ser158Leu) (Table 1). Based on the findings of severely elevated plasma oxalate concentration (pre-hemodialysis, 323 μmol/L; post-hemodialysis 68.62 μmol/L) and CaOx crystals involving the renal graft (Fig. 1B), the patient was diagnosed with PH1. Conservative treatments were administered, including pyridoxine (vitamin B6), and continuous renal replacement therapy followed by temporarily intensive hemodialysis to reduce plasma oxalate concentration. However, his renal function (Fig. 2) and CaOx crystal deposition in the renal graft gradually worsened (Fig. 1D). Compared with preoperative CT images, images at 4 and 7 weeks after surgery showed that the coronary calcification of the heart has worsen (Supplementary Figure 1). Based on his condition, we recommended that the patient receive a liver transplantation first, and then receive kidney transplantation based on the recovery status of the patient’s renal function. On May 11th, the patient underwent living-donor liver transplantation performed utilizing the right lobe and bilateral nephrectomy, considering that high concentrations of plasma oxalate can continue to affect the function of the renal graft (Supplementary Figure 2). Eight weeks after liver transplantation, the patient’s renal function did not improve, and his SCr was 671.00 μmol/L. However, his plasma oxalate concentration decreased to 195.4 μmol/L (pre-hemodialysis). The patient received the maintenance hemodialysis with looking forward to a chance of second kidney transplantation in the future.

**Table 1:** AGXT gene mutations identified in the family of patient

	Age	AGXT gene		Kidney stones	Renal insufficiency
		exon1/CDS1: c .120-21ins (p.Gly41Argfs*127)	exon4/CDS4: c.473C > T (p.Ser158Leu)		
Patient	33Y	**+**	**+**	**+**	**+**
Father	59Y	**–**	**–**	**–**	**–**
Mother	57Y	**+**	**+**	**+**	**–**

The MEDLINE/PubMed, Embase, Web of Science, and CNKI databases were searched in April 2021 for all articles from 2000 to 2021, with English or Chinese language limits applied. A total of 19 cases of PH diagnosed after kidney transplantation, including 18 cases from the databases and our case, were collected and reviewed (Table 2). Patients were mainly reported in developing countries with a median age of 34 years. Among these 19 cases, 11 cases (57.9%) are PH 1, and 4 cases (21.1%) are PH 2. It is worth noting that approximately 78.9% of the patients (15/19) had a history of bilateral nephrolithiasis. All studies reported that deposition of oxalate crystals could be detected by biopsy of the renal graft as early as 5 days after transplantation. In all patients who underwent biopsy within 1 month after transplantation, only two cases (2/8) had borderline acute rejection, and no cases of acute T-cell mediated rejection (TCMR) were reported. However, five acute TCMR cases (5/11) and one borderline acute rejection case (1/11) were detected from biopsies taken more than 1 month post-transplantation. A total eight cases (42.1%) were misdiagnosed as having acute rejection before needle biopsy. In all of these cases, postoperative complications included fever, viral infections, diarrhea, oral candidiasis, necrotizing skin lesions, abdominal pain, breathlessness, and urinary tract infection. After receiving conservative treatment, only three patients (3/19) reported no further deterioration of renal function; however, most of the patients (14/19) had progressive deterioration or eventually received hemodialysis.

**Table 2:** Clinical data of reported cases of PH diagnosed after renal transplantation

Study	Country	Age (year)	Gender	Type of PH	Bilateral nephrolithiasis	Time of biopsy after transplantation	Biopsy of renal graft	Misdiagnosed as acute rejection	Outcomes
Riksen et al. (2002) [5]	Yugoslavia	51	Male	PH1	Yes	64 days	OCD + No signs of rejection	Yes	no deterioration (8 months)
Kim et al. (2005) [6]	Korea	43	Female	PH1	No	11 days	OCD + No signs of rejection	Yes	progressive deterioration
Zhu et al. (2005) [7]	China	34	Male	PH1	Yes	2 months	OCD + No signs of rejection	Yes	maintenance HD
Madiwale et al. (2008) [8]	India	25	Male	Unknown	Yes	5 days	OCD + No signs of rejection	Yes	death (10 weeks)
Chen et al. (2008) [9]	China	47	Female	Unknown	Yes	7 months	OCD + No signs of rejection	Yes	Unknown
Celik et al. (2010) [10]	Turkey	38	Male	Unknown	Yes	13 days	OCD + No signs of rejection	No	no deterioration (60 months)
Spasovski et al. (2010) [11]	Macedonia	48	Female	PH1	No	3 weeks	OCD + borderline acute rejection	No	maintenance HD
Malakoutian et al. (2011) [12]	Iran	22	Female	PH1	Yes	2 months	OCD + No signs of rejection	Yes	maintenance HD
Naderi et al. (2015) [13]	Iraq	20	Male	PH1	Yes	10 days	OCD + No signs of rejection	Yes	maintenance HD
Wang et al. (2016) [14]	China	32	Male	PH2	Yes	26 days	OCD + borderline acute rejection	No	maintenance HD
Rios et al. (2017) [15]	Colombia	33	Female	PH1	Yes	1 year	OCD + acute TCMR (Banff 1A)	No	progressive deterioration
	Colombia	53	Unknown	PH2	Yes	4 months	OCD + acute TCMR (Banff 2A)	No	maintenance HD
Liu et al. (2018) [16]	China	33	Male	PH2	Yes	15 days	OCD + No signs of rejection	No	maintenance HD
Cai et al. (2019) [17]	China	27	Male	PH2	Yes	46 days	OCD + acute TCMR (Banff 1A)	No	maintenance HD
	China	26	Male	PH1	Unknown	38 days	OCD + acute TCMR (Banff 2A)	No	maintenance HD
	China	34	Male	Unknown	No	75 days	OCD + acute TCMR (Banff 2A)	No	maintenance HD
Zhao et al. (2020) [18]	China	52	Male	PH1	Yes	2 months	OCD + borderline acute rejection	No	no deterioration (36 months)
Wang et al. (2021) [19]	China	57	Female	PH1	Yes	4 months	OCD + No signs of rejection	Yes	maintenance HD
Current Study	China	32	Male	PH1	Yes	21 days	OCD + borderline acute rejection	No	maintenance HD

*OCD* oxalate crystals deposition, *PH* measure primary hyperoxaluria, *TCMR* T cell mediated rejection, *HD* hemodialysis

## Discussion and conclusion

There are various manifestations of systemic oxalosis due to PH1; however, it mainly manifests as recurrent urolithiasis, nephrocalcinosis and end-stage renal disease. In the 19 cases reviewed, PH diagnosis after DGF mainly occurred in developing countries. Due to the lack of understanding of PH disease, history of recurrent nephrolithiasis and the calcification of both kidneys are often ignored, which leads to many patients failing to receive a clear diagnosis of PH before undergoing transplantation. In addition, because many patients do not undergo kidney biopsy before transplantation and laboratory examination for plasma or urine oxalate concentration is not routinely performed in many hospitals in developing countries, the chance of being diagnosed with PH before kidney transplantation is reduced. For these reasons, unfortunately DGF occurs in many patients due to PH recurrence. In our study, considering that the calcification of the kidneys and the thinning of the renal cortex may increase the risk of bleeding and infection after renal biopsy, pretransplant renal biopsy has not been performed. However, this makes us miss the chance to diagnose PH1 before transplantation.

Considering that not all patients are willing to receive renal biopsy and molecular genetic testing before kidney transplantation, it is necessary to understand the manifestations of PH to facilitate careful pre-transplant PH screening. To the best of our knowledge, there are 18 cases reported cases of PH which were diagnosed after kidney transplantation. All of these patients underwent renal transplantation biopsy for the diagnosis of PH due to early transplanted kidney loss. After reviewing all reported cases and our own case, we found that 78.9% of these patients had a history of bilateral nephrolithiasis. The remaining reported organs with deposits of oxalate crystals included the heart, pancreas, skin, lungs, spleen, coronary vessels and bone marrow. Compared with preoperative CT images, images after surgery showed that the coronary calcification of the heart has worsen. However, as cardiac biopsy or surgery was not performed, we can only speculate that oxalate crystals had precipitated in the coronary vessels of the heart. We believe that patients with bilateral nephrolithiasis, or bilateral kidney stones with multiple organ calcification should be routinely checked for oxalate levels before transplantation, and genetic screening should be performed if indicated.

Conservative treatments (including high fluid intake, avoidance of high oxalate foods, and vitamin B6) should be initiated as soon as PH is diagnosed before transplanting. A high fluid intake (at least 3 L/m2 per 24 h), can reduce urinary oxalate supersaturation, and this has proven to be effective in previous studies [20]. Pyridoxine (vitamin B6) is a precursor to pyridoxal-5-phosphate which acts as a cofactor of AGT by increasing the transamination of glyoxylate (a precursor of oxalate) to glycine [21]. The molecular mass of oxalic acid is 90 Da, and dialysis can be partially effective in handling the oxalate load. However, standard hemodialysis can eliminate 6–9 mmol/1.73m^2^ of oxalate per week, which is less than half of the weekly endogenous production of oxalate [22]. Compared with low-flux filters, high-flux filters have a slight advantage in terms of eliminating oxalate [23]. Therefore, intensified hemodialysis with more frequent and longer sessions is more efficient than standard hemodialysis in maintaining plasma oxalate levels below the level of oversaturation of plasma CaOx [24]. Alkali citrate can form complexes with calcium and decrease the saturation of CaOx in the urine. The above conservative treatments before and after transplantation can decrease the plasma oxalate concentration and reduce the damage to the renal graft and other organs. Isolated kidney transplantation should not be considered, because it doesn’t solve the problem of deficiency of enzyme for glyoxylate metabolism [22]. For patients who have developed ESRD, combined liver and kidney transplant is an optimal modality of treatment [25]. Sequential transplant (transplant of liver and kidney at different time periods) may be proposed in individual ESRD patients. After combined liver-kidney transplant or sequential transplant, urine oxalate can remain elevated due to resolubilization of CaOx in tissues slowly. Therefore, fluid intake, crystallization inhibitors should be used to protect the renal graft.

Currently, conservative treatments are unsatisfactory for the treatment of DGF secondary to PH [26]. Three patients with PH reported a reversal of the continuous increase in creatinine by oral vitamin B6 therapy [5, 10, 18]. This may be due to the fact that some patients with specific mutations in AGXT are sensitive to B6 treatment [18, 27]. To the best of our knowledge, this is the first case to report the plasma oxalate concentration of pre-continuous and post-continuous renal replacement therapy (72 h) in a patient with PH diagnosed after kidney transplantation. These results suggest that hemodialysis can significantly reduce plasma oxalate concentration, indicating that early hemodialysis may be helpful in reducing oxalate crystal deposition in organs and improving the early survival of patients after surgery. However, hemodialysis cannot completely solve the problem of continued deposition of CaOx in important organs. Currently, liver transplantation is the only way to cure PH [28]. Our patient underwent a liver transplantation 3 months after loss of renal allograft function. Although the plasma oxalate level decreased 2 weeks after liver transplantation, the transplanted kidney function did not improve. However, further follow-up is needed to evaluate the overall effects of liver transplantation.

In all 19 cases, the postoperative complications included fever, viral infections, diarrhea, and urinary tract infection. It is worth noting that patients with PH have a high risk of postoperative infection. We believe that the risk of postoperative infection may be related to enhanced immunosuppression due to the misdiagnosis of acute rejection. The DGF caused by PH is easily misdiagnosed as acute rejection, therefore, needle biopsy should be performed early to reduce the duration of enhanced immunosuppressive applications. However, most patients included in this literature review were from individual case reports because of the rarity of this disease. Large-scale, multi-center research on patients receiving kidney transplantation with PH is needed for further research.

In summary, our case reports and literature reviews show that the proportion of kidney transplant failure caused by a missed diagnosis of PH before kidney transplantation is extremely high. Preoperative PH screening, including urine oxalate analysis, molecular genetic testing and renal biopsy, should be performed in all patients with bilateral nephrocalcinosis or nephrolithiasis.

## Supplementary Information

**Additional file 1: Supplementary Figure 1.** CT images before and after transplantation showed that the coronary calcification of the heart has worsen

**Additional file 2: Supplementary Figure 2.** CT images before and after liver transplantation and bilateral nephrectomy

## Data Availability

The raw data that support the findings of this report are available from Renmin Hospital of Wuhan University.

## Abbreviations

PH: Primary hyperoxaluria
AGXT: Alanine-glyoxylate aminotransferase
GRHPR: Glyoxalate reductase/hydroxy pyruvate reductase
HOGA: 4-hydroxy-2-oxoglutarate aldolase
ESRD: End-stage renal disease
DGF: Delayed graft function
SCr: Serum creatinine
MMF: Mycophenolate mofetil
TCMR: T cell mediated rejection
