# Supplementary material for: Primary hyperoxaluria diagnosed after kidney transplantation: a case report and literature review
Source: BMC Nephrol. 2021 Nov 27;22:393. doi: 10.1186/s12882-021-02546-0 (PMC8626922; doi:10.1186/s12882-021-02546-0)
Supplement: Supplementary file 1 — Additional file 1: Supplementary Figure 1. CT images before and after transplantation showed that the coronary calcification of the heart has worsen [file 12882_2021_2546_MOESM1_ESM.pdf]

Supplementary Figure 1

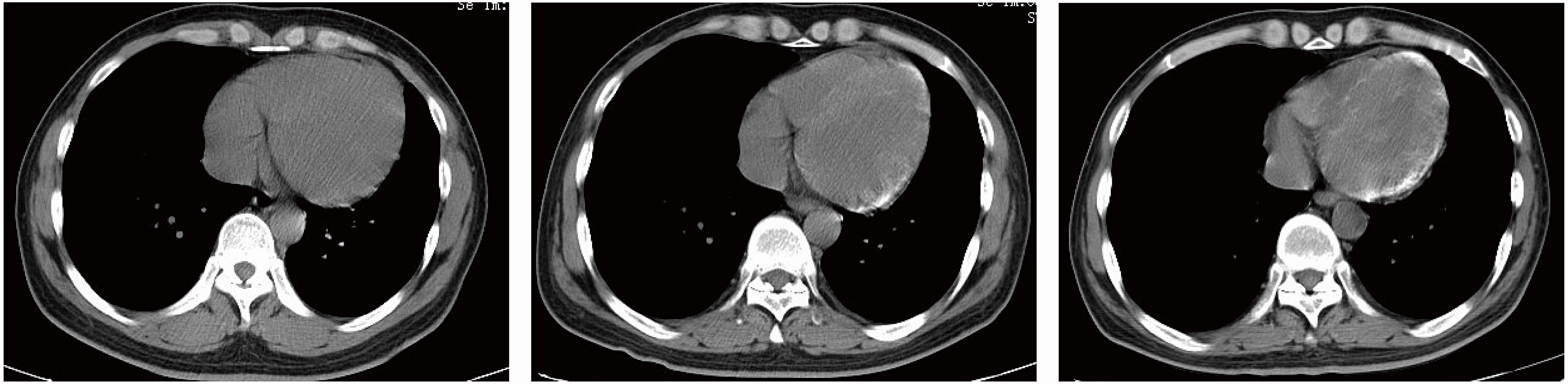

Before transplantion

4 weeks after transplantion

7 weeks after transplantion
