# Supplementary figures and images for: Primary hyperoxaluria diagnosed after kidney transplantation: a case report and literature review
Source: BMC Nephrol. 2021 Nov 27;22:393. doi: 10.1186/s12882-021-02546-0 (PMC8626922; doi:10.1186/s12882-021-02546-0)

## Supplementary Figure 2

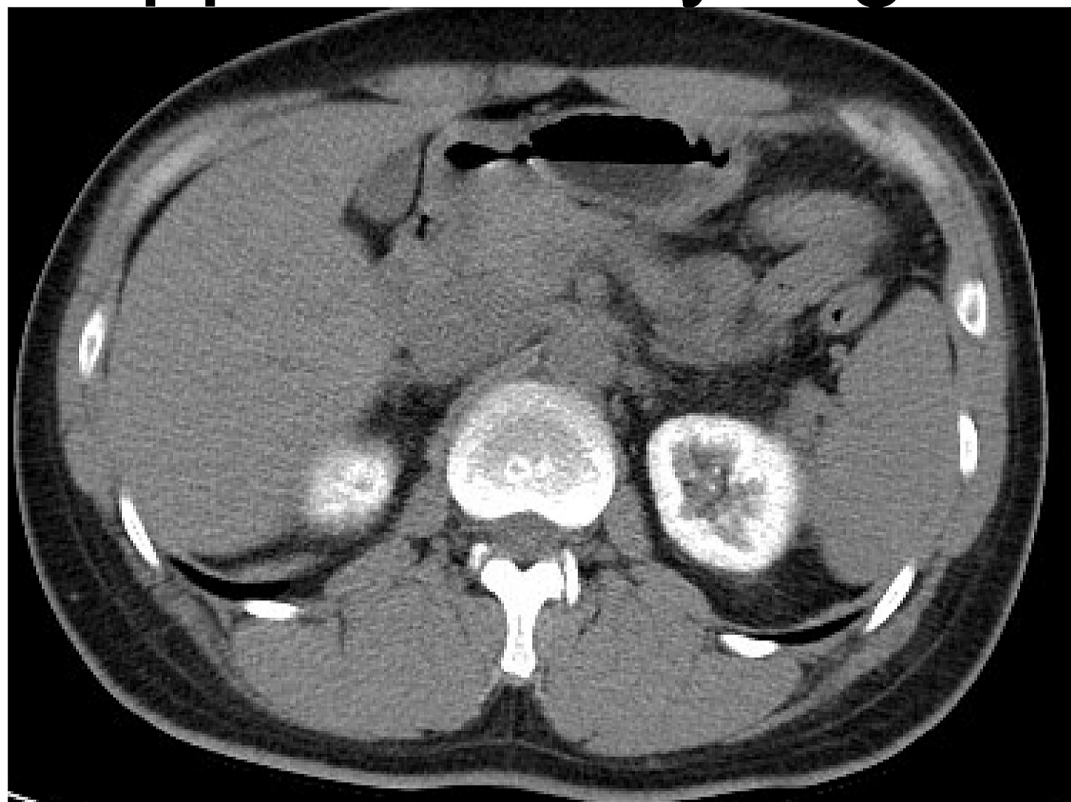

Before surgery

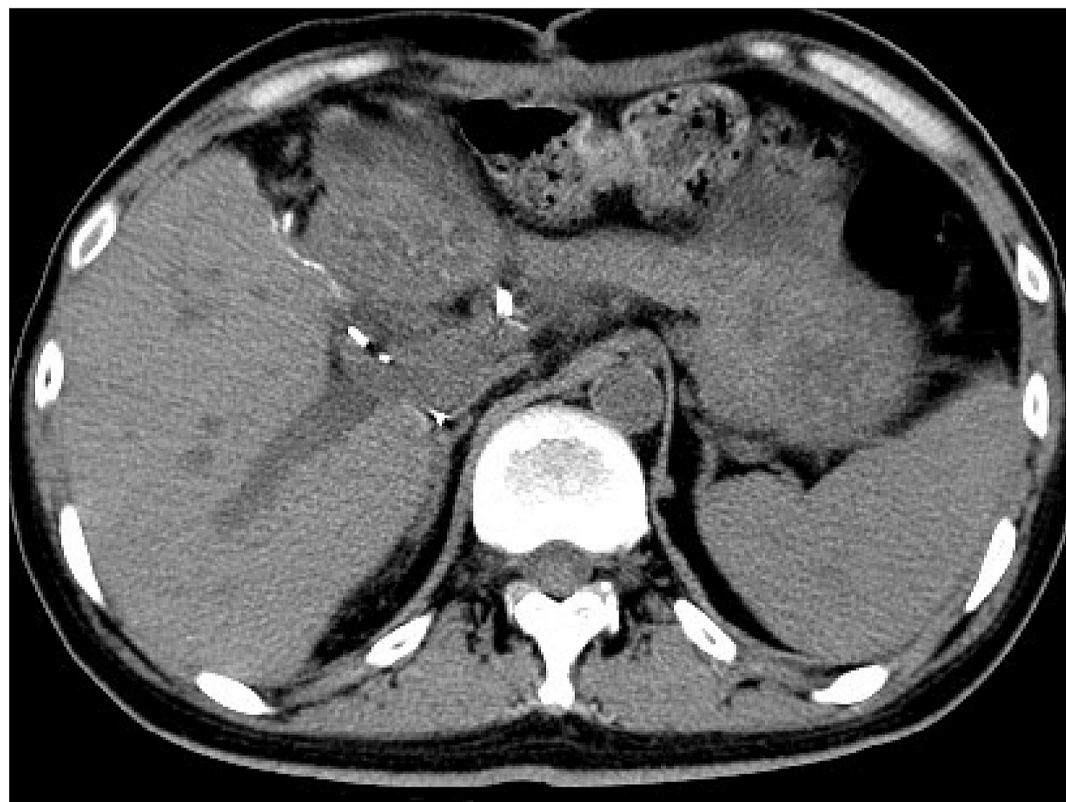

2 months after surgery

Supplement: Supplementary file 2 — Additional file 2: Supplementary Figure 2. CT images before and after liver transplantation and bilateral nephrectomy [file 12882_2021_2546_MOESM2_ESM.pdf]
